# Clonal haematopoiesis of indeterminate potential: intersections between inflammation, vascular disease and heart failure

**DOI:** 10.1042/CS20200306

**Published:** 2021-04-16

**Authors:** Leanne Mooney, Carl S. Goodyear, Tamir Chandra, Kristina Kirschner, Mhairi Copland, Mark C. Petrie, Ninian N. Lang

**Affiliations:** 1 BHF Glasgow Cardiovascular Research Centre, University of Glasgow, Glasgow, U.K.; 2 Institute of Immunity, Infection and Inflammation, University of Glasgow, Glasgow, U.K.; 3 The Institute of Genetics and Molecular Medicine, University of Edinburgh, Western General Hospital, Crewe Road, Edinburgh, U.K.; 4 Paul O’Gorman Leukaemia Research Centre, Institute for Cancer Science, University of Glasgow, Glasgow, U.K.

**Keywords:** ageing, atherosclerosis, cardiovascular disease, clonal haematopoiesis of indeterminate potential, heart failure

## Abstract

Ageing is a major risk factor for the development of cardiovascular disease (CVD) and cancer. Whilst the cumulative effect of exposure to conventional cardiovascular risk factors is important, recent evidence highlights clonal haematopoiesis of indeterminant potential (CHIP) as a further key risk factor. CHIP reflects the accumulation of somatic, potentially pro-leukaemic gene mutations within haematopoietic stem cells over time. The most common mutations associated with CHIP and CVD occur in genes that also play central roles in the regulation of inflammation. While CHIP carriers have a low risk of haematological malignant transformation (<1% per year), their relative risk of mortality is increased by 40% and this reflects an excess of cardiovascular events. Evidence linking CHIP, inflammation and atherosclerotic disease has recently become better defined. However, there is a paucity of information about the role of CHIP in the development and progression of heart failure, particularly heart failure with preserved ejection fraction (HFpEF). While systemic inflammation plays a role in the pathophysiology of both heart failure with reduced and preserved ejection fraction (EF), it may be of greater relevance in the pathophysiology of HFpEF, which is also strongly associated with ageing. This review describes CHIP and its pathogenetic links with ageing, inflammation and CVD, while providing insight into its putative role in HFpEF.

## Introduction

**Figure 1:**
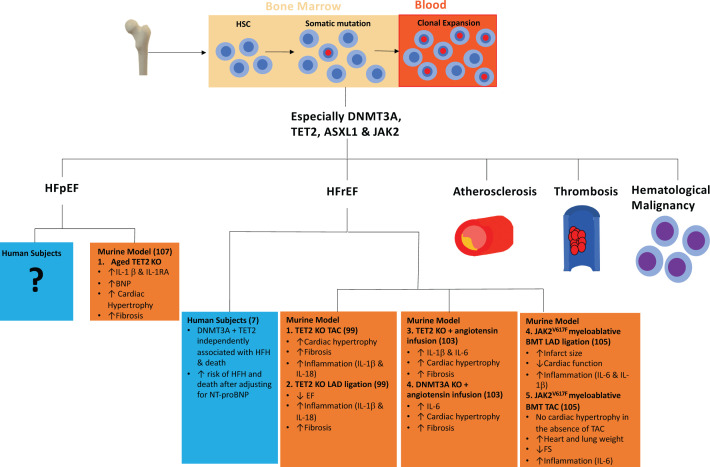
Development of clonal haematopoiesis, associated risk factors and its role in heart failure (murine and humans) Abbreviations: HSC, haematopoietic stem cells; EF, ejection fraction; FS, fractional shortening; HFH, heart failure hospitalisation; HFrEF, heart failure with reduced ejection fraction; KO, knockout; LAD, left anterior descending artery; NT-proBNP, B-type natriuretic peptide; TAC, transverse aortic constriction.

Cardiovascular disease (CVD) and cancer are the two leading causes of deaths worldwide and ageing is a major risk factor for the development of both of these major disease processes [1,2]. To a large extent, these age-associated risks reflect the cumulative effects of exposure to ‘conventional’ shared risk factors such as smoking and obesity. However, *clonal haematopoiesis of indeterminate potential (CHIP)* may provide a further important link, particularly in the pathogenesis of CVD [3]. CHIP, also known as *age-related clonal haematopoiesis (ARCH)*, reflects the accumulation of potentially pre-leukaemic, somatic mutations in haematopoietic stem cells (HSCs) over time [3,4]. However, whilst the risk of malignant transformation of CHIP is low, its presence confers a substantially greater risk of CVD (Figure 1) [3,5–9].

**Figure 2:**
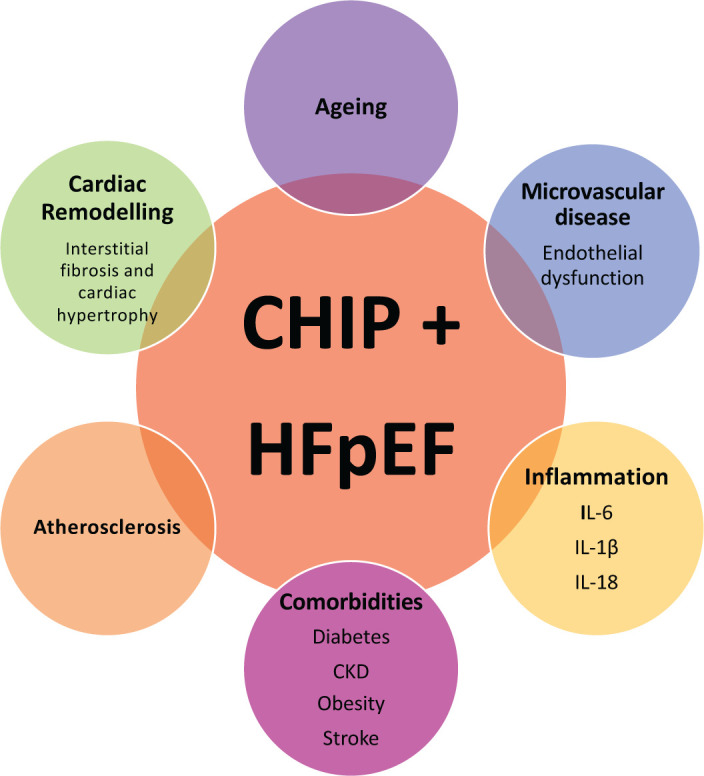
Potential mechanistic links between CHIP and HFpEF Abbreviations: CKD, chronic kidney disease; HFpEF, heart failure with preserved ejection fraction; IL-6, interleukin-6; IL-18, interleukin 18; IL-1β, interleukin-1β.

In this review, we will provide a primer on CHIP and its pathogenetic links with ageing, inflammation and CVD. In particular, we provide a framework to inform further investigation of the role of CHIP as a risk factor and pathogenetic mediator in patients with heart failure, especially in heart failure with preserved ejection fraction (HFpEF).

## CHIP: a primer

### Definition and overview of CHIP

Current diagnostic criteria for clonal haematopoiesis of indeterminate potential (CHIP) include: (1) the absence of overt haematological malignancy; (2) a normal peripheral blood count and (3) mutant cells bearing relevant driver mutations in ≥2% of peripheral white blood cells (variant allele frequency [VAF] ≥ 2%) [10]. By the age of 70 years, 10–20% of the otherwise healthy population have a peripheral blood leucocyte clone with a VAF of at least 2% and meet the criteria for CHIP [5,9,11–13]. Conversely, CHIP is found in fewer than 1% of patients under the age of 50 years [3,9,13].

CHIP can be detected via DNA sequencing of peripheral blood, saliva and tumour samples and, while deep-sequencing methods may detect a VAF of less than 2%, the clinical consequences of these smaller clonal populations are unknown [14–16]. The majority of these age-associated mutations are cytosine (C) to thymine (T) transitions, consistent with the signature of mutations seen across many different types of cancer [3,9]. The most frequently encountered somatic mutations are within the driver genes ten-eleven-translocation-2 (TET2), DNA methyltransferase 3 [DNMT3]), Janus kinase 2 (Jak2) and additional sex comb-like 1 (ASXL1). CHIP-associated mutations are also found, albeit less frequently, in other driver genes outlined in (Table 1) [17].

**Table 1:** Most frequent somatic mutations in CHIP

Gene	Name	Description
*TET2*	Ten-eleven-translocation-2	A methylcytosine dioxygenase that catalyses the conversion of 5-methylcytosine into 5-hydroxymethylcytosine. An epigenetic regulator that can activate or repress transcription.
*DNMT3A*	DNA methyltransferase 3A	A *de novo* DNA methyltransferase.
*Jak2*	Janus kinase 2	Receptor tyrosine kinase involved in haematopoietic cytokine signalling and myelopoiesis.
ASXL1	Additional sex comb-like 1	Polycomb chromatin-binding protein that is involved in the transcriptional regulation of *Hox* genes.
*PPMD1*	Protein phosphatase, magnesium/manganese- dependent 1D	Protein phosphatase involved in dephosphorylation and inactivation of proteins in the DNA damage response pathway.
*SF3B1*	Splicing factor 3B, subunit 1	A component of the U2 small nuclear riboprotein that binds to the 3′ branch site in pre-mRNA splicing and processing.
*SRSF2*	Serine/Arginine-rich splicing factor 2	Required for 5′ and 3′ spliceosome assembly, splice-site selection, U1 and U2 snRNP interactions with pre-mRNA, and alternative splicing.
*TP53*	Transformation-related protein 53	Tumour suppressor transcription factor that responds to cellular stress and DNA damage.

Adapted from [11]. Abbreviations: DNA, deoxyribonucleic acid; mRNA, messenger ribonucleic acid; snRNP, small nuclear ribonucleioprotein.

### Triggers and risk factors for CHIP

Little is known about the triggers to clone initiation and expansion. The natural process of ageing results in an increased likelihood of retaining somatic mutations [18,19]. At the molecular level, DNA damage, telomere shortening and autophagy appear to be central mechanisms underlying age-related functional impairment and decline in the durability of HSCs [20,21]. Chronic low-grade inflammation occurs with ageing (recently described as *inflammageing*) and may also be partly responsible [22]. Indeed, exposure of mice to the pro-inflammatory mediator, tumour necrosis factor-α (TNF-α), promotes the expansion of TET2 mutant clones and exposure to inflammatory stress in myeloid cells results in the rapid increase in frequency and absolute number of TET2-mutated myeloid cells [23,24]. Exogenous stressors that directly provoke inflammation, DNA damage, telomere shortening and production of reactive oxygen species may lead to the premature exhaustion of HSCs and an increased likelihood of retaining somatic mutations at a younger age [25]. Consistent with this hypothesis, prior chemotherapy and radiotherapy are associated with an increased susceptibility to the retention of these somatic mutations in humans [26,27]. To date, little attention has been paid to the environmental factors that may influence the development of CHIP but smoking, diet and diabetes have been associated with risk for clonal expansion in humans [3,13,28] (Table 2). While there has been speculation about a potentially heritable risk of CHIP, this was not confirmed in studies of mono- and di-zygotic twins [29,30].

**Table 2:** Risk factors for CHIP

	Degree of risk	References
**Non-modifiable risk factors**		
Age	↑	[3,9,13]
Male sex	↑	[3]
**Race**		
Hispanic ancestry	↓	[3]
Asian ancestry	↓	[34]
**Modifiable risk factors**		
Smoking	↑	[13]
Diabetes	↑	[3]
Unhealthy diet	↑	[28]
**Radiation exposure**	↑	[26]
**Chemotherapy exposure**		
Platinum agents (cisplatin, carboplatin and oxaliplatin)	↑	[26,157]
Topoisomerase inhibitors (e.g. etoposide)	↑	

### CHIP and risk for haematological malignancy and CVD

CHIP belongs to a spectrum of haematological pre-malignant states and is associated with the development of various haematological malignancies including leukaemia, lymphoma and myeloma [3,26]. However, most carriers will not develop malignancy and the progression rate is approximately 0.5–1% per year [31]. Malignant transformation or progression generally requires the acquisition of multiple mutations and directly correlates with the mean VAF [3,10]. It is notable that patients found to have CHIP at the time of autologous stem cell transplantation are at an increased risk for the subsequent development of therapy-related myeloid neoplasm (myelodysplastic syndrome and acute myeloid leukaemia) [32].

Despite the low risk of progression to haematologically important diagnoses, all-comers with CHIP have a 40% higher mortality than those without CHIP, and this striking excess is a reflection of cardiovascular events [3]. The presence of CHIP confers a substantially increased risk for CVD independent of traditional risk factors including diabetes and hypercholesterolaemia [3,5].

### CHIP and inflammation

The effects of specific CHIP-associated mutations are yet to be fully described, but a core feature appears to be the establishment of a pro-inflammatory state. Compared with those without CHIP, people with evidence of clonal haematopoiesis have higher circulating concentrations of pro-inflammatory markers including interleukin-6 (IL-6), TNF-α and monocyte chemoattractant protein 1 (MCP-1) [33,34]. Driver gene-specific analysis of a large cohort of individuals with CHIP highlighted the association of TET2 mutations with increased IL-1β, whereas Jak2 and SF3B1 mutations were associated with higher circulating IL-18 [34]. Other, potentially less sensitive markers of inflammation such as white blood count (WBC), neutrophil count, C-reactive protein (CRP) and erythrocyte sedimentation rate (ESR) are not normally elevated in people with CHIP [33,34]. It has been proposed that the role of inflammation in CHIP is bidirectional, whereby inflammation initially predisposes to the development of CHIP, with consequent unregulated pro-inflammatory cytokine release via a feedback loop [35]. Of the mutations associated with CHIP, TET2, DNMT3A, Jak2^V617F^ and ASXL1 are the most frequent. To date, it is unknown whether specific mutations in the TET2, DNMT3A and ASXL1 genes have different clinical consequences. Several different mutations have been reported to occur in each gene and the pathophysiologic effects of these have not yet been individually characterised [3,10].

### TET2

Mutation of TET2 was the first somatic genetic abnormality to be reported in blood cells from individuals with clonal haematopoiesis without overt haematological malignancy [36]. TET2 is a member of a family of enzymes located on chromosome 4q23 and is an epigenetic regulator of DNA methylation. It catalyses the oxidation of 5-methylcytosine (5mC) to 5-hydroxymethylcystosine (5hmC) as the first step in cytosine demethylation [37]. This activity is critical for maintaining the normal development of HSCs. TET2 mutations are loss-of-function mutations associated with a decrease in 5hmC availability and consequently this has been proposed as a potential diagnostic and prognostic biomarker in haematological malignancy [38]. Whether it holds the same potential utility in the prediction of CHIP/TET2 mutation-associated CVD remains to be tested. TET2 also plays an important role in the regulation of the immune system, and evidence suggests that TET2-mediated clonal haematopoiesis contributes to the pathophysiology and progression of CVD through its induction of a pro-inflammatory state. TET2 controls the secretion of pro-inflammatory cytokines through modulation of histone acetylation [39,40]. Lipopolysaccharide (LPS) and interferon-γ (IFN-γ) stimulation of macrophages from TET2 deficient mice induces the hyperactivation of pro-inflammatory cytokines and chemokines including IL-1β and IL-6 [39]. Furthermore, loss of TET2 in myeloid-derived cells results in a higher expression of IL-6 in mice [40]. In an unselected cohort of patients without CVD, the presence of TET2 mutation was associated with over two-fold higher circulating concentrations of IL-8 than in those without this mutation [5].

### DNMT3A

DNMT3A modulates gene transcription via the catalysis of DNA methylation and is the most frequently mutated gene in people with CHIP. DNMT3A mutations are thought to be loss-of-function mutations although there are reports that some mutations may lead to gain-of-function, conferring increased HSC self-renewal and subsequent clonal expansion [41,42]. DNMT3A also has multiple roles in the regulation of inflammation. In particular, it controls cytokine expression through the regulation of the scaffold protein IQ motif containing GTPase Activating Protein 2 (IQGAP2) in mast cells [43]. In patients with osteoarthritis, IL-6 gene activity is associated with the expression of DNMT3A and significantly lower levels of IL-6 secretion are found in those with DNMT3A overexpression [44]. Furthermore, in patients with severe aortic stenosis, the presence of DNMT3A mutations has been associated with significantly elevated T helper 17 cell (TH17): regulatory T cells (Tregs) ratio, representing pro-inflammatory T-cell polarisation [8].

### Jak2^V617F^

Of CHIP-associated genetic abnormalities, Jak2^V617F^ gain-of-function mutation has been linked most clearly to inflammatory processes. In humans, it serves as a signal transmitter downstream of major cytokine receptors resulting in activation of granulocytes, T cells, enhanced inflammation in macrophages and activation of neutrophil extracellular traps [45]. V617F somatic mutation of the Jak2 gene reflects substitution of phenylalanine for valine at position 617. Jak2^V617F^ mutations are commonly associated with myeloproliferative neoplasms including essential thrombocythaemia (ET) and polycythaemia vera (PV) [46]. These conditions are associated with an increased risk of stroke, myocardial infarction and deep vein thrombosis, primarily as a result of increased blood viscosity and a pro-coagulant state. However, Jak2^V617F^ mutations are increasingly recognised in individuals with normal peripheral blood counts, and remain associated with increased cardiovascular mortality [13,47–49].

### ASXL1

ASXL1 encodes an epigenetic regulator which binds to chromatin. It is one of the most frequently mutated genes in myeloid neoplasms and its presence is associated with poor prognosis [50–52]. The majority of mutations are frameshift or nonsense mutations and frequently coexist with TET2, IDH1 and IDH2 mutations [52–55]. However, whether these truncations of the protein lead to loss- or gain-of-function remains controversial [56–58]. Mutation of ASXL1 is common in patients with atherosclerosis and chronic ischaemic heart failure but the mechanisms contributing to this increased CV risk are not defined [5,7].

## CHIP and vascular disease

Atherosclerosis is an inflammatory disease, predominantly of the macro-vasculature. Almost 60% of elderly patients with atherosclerosis have either no conventional risk factors (e.g. hypertension or hypercholesterolaemia) or have only one risk factor, thus implying the presence of otherwise unidentified predisposing conditions [59]. CHIP has been identified as a potential factor closely linked to the initiation and progression of atherosclerosis [5]. Microvascular disease involves a complex interplay between upstream atherosclerosis, inflammation and endothelial dysfunction. Of the CHIP-related mutations, the role of TET2 has been most clearly defined in relation to vascular disease and normal TET2 function has been implicated in several important regulatory processes in both the macro- and microcirculation [60–64]. These include suppression of vascular smooth muscle cell (VSMC) phenotypic transformation, protective effects upon endothelial cells as well as anti-inflammatory and anti-atherogenic effects [60–64].

### CHIP and human atherosclerosis

Nested case–control analyses of prospective cohorts, that together enrolled 4726 participants with coronary artery disease and 3529 controls, revealed that carriers of CHIP (DNMT3A, TET2 and ASXL1 mutations) have a risk of coronary artery disease that is substantially greater than controls (Table 3). Indeed, patients with CHIP were twice as likely to have a history of myocardial infarction or coronary revascularisation than people without CHIP [5]. CHIP-associated DNMT3A mutation was associated with a hazard ratio of 1.7 for coronary artery disease while TET2 mutation conferred a hazard ratio of 1.9. Those with Jak^V617F^ mutation had the highest increased risk of coronary artery disease, which was 12-times greater than people with no mutation. In younger patients, the association between CHIP and atherosclerotic risk was even stronger than in older individuals [5]. In the same study, people with CHIP without a prior diagnosis of coronary artery disease were three times more likely to have a computed tomography (CT) coronary artery calcification (CAC) score of at least 615 Agatston units [5], the empirical cutoff for the identification of older patients at high risk of coronary events [65]. This coronary artery calcification score correlated positively with percentage VAF implying a ‘dose effect’ of the accumulation of mutated cells. Patients with large mutant clone populations (VAF > 10%) without a prior diagnosis of coronary artery disease were 12-times more likely to have a CAC score over 615 Agatston units [5]. In a large genome-wide association study, the presence of CHIP-associated Jak2 mutation was associated with increased risk of coronary artery disease despite lower levels of triglycerides and low-density lipoprotein (LDL) cholesterol [66].

**Table 3:** CHIP mutations and associated cardiovascular risk

Cohort	Mutation	Age	Cardiovascular risk	HR	Ref
US population based	Any CHIP mutation	Median 58 years	Incident coronary artery disease	2.0 (1.2–3.5)	[3]
			Ischaemic stroke	2.6 (1.3–4.8)	
PROMIS	Any CHIP mutation	<50 years	Early onset myocardial infarction (before the age of 50)	4.0 (2.4–6.7)	[5]
ATVB				5.4 (2.3–13.0)	
U.K. Biobank	Any CHIP mutation	Mean 61 years	Myocardial infarction, coronary artery revascularisation, stroke or death	1.27 (1.04–1.56)	[6]
Chronic ischaemic HFrEF	TET2 or DNMT3A	Median 69 years	Heart failure hospitalisation or all-cause death	2.1 (1.1–4.0)	[7]
Severe aortic stenosis undergoing transcatheter aortic valve replacement	TET2 or DNMT3A	Median 83 years	Risk of death following transcatheter aortic valve replacement	3.1 (1.17–8.08)	[8]

Abbreviations: ATVB, Atherosclerosis, Thrombosis, and Vascular Biology Italian Study Group; HFrEF, heart failure with reduced ejection fraction; HR, hazard ratio; PROMIS, Pakistan Risk of Myocardial Infarction Study.

Endothelial dysfunction is the earliest feature in the development of atherosclerosis. Patients with coronary endothelial dysfunction (assessed via vasomotor responses to intra-coronary acetylcholine infusion) have significantly higher prevalence of CHIP-associated mutations in comparison with people with normal coronary endothelial function (9.2 versus 1.5%, respectively) [67]. Furthermore, somatic mutations in ASXL1, DNMT3A and TET2 are associated with higher levels of IL-6 and IL-8 in this group [67].

The potential association between CHIP, inflammation and CVD was assessed in 35416 people included in the U.K. Biobank (Table 3) [6]. Participants did not have a history of CVD at inclusion but those with DNMT3A or TET2 mutation had a 27% higher risk of CVD over 6.9 years of follow-up when compared with those without these CHIP mutations [6]. This risk was larger in those with larger clones denoted by VAF >10% (hazard ratio 1.59 [95% CI: 1.21–2.09]) [6]. Furthermore, to examine the potential interaction with inflammation, the effect of carrying a genetic proxy of IL-6 inhibition (IL6R p.Asp358Ala) and simultaneous CHIP was also assessed [6]. In people with large CHIP clones (VAF > 10%), the presence of this genetic proxy was associated with a 54% lower risk of CVD events and was without effect upon CVD event risk in individuals without CHIP [6]. In those aged over 50 years with a history of prior myocardial infarction and CHIP, each additional IL6R p.Asp358Ala allele attenuated the risk of CVD events [6]. Not only do these genetic data provide further mechanistic insight concerning interactions between CHIP, inflammation and CVD, they also give weight to the hypothesis that therapeutic inhibition of IL-6 signalling may prove to be beneficial in patients with large CHIP clones and CVD.

### TET2 – preclinical vascular models

The first murine model to implicate the role of CHIP in atherosclerosis aimed to mimic human clonal haematopoiesis by initially introducing a small number of mutant TET2 cells. This model used a competitive bone marrow transplantation strategy to generate atherosclerotic prone, LDL receptor deficient (Ldlr^−/−^) chimeric mice with a small proportion of TET2-deficient HSC (10% TET2^−/−^ bone marrow) [68]. Importantly, when compared with control mice, there was no difference in body weight, plasma cholesterol levels, glucose and systemic insulin sensitivity. Following nine weeks of a high fat/high cholesterol diet, TET-2 deficient mice (10% knockout [KO]-BMT) developed aortic root plaques that were 60% larger than those of control animals. This increased atherogenesis in 10% KO-BMT mice was paralleled by an increase in total macrophage content in the intima, and these TET2-deficient macrophages exhibited markedly increased expression of pro-inflammatory cytokines. In particular, the transcription of aortic arch IL-1β in macrophages was doubled and treatment with the nucleotide-binding domain leucine-rich repeat containing receptor 3 (NLRP3) inflammasome inhibitor, MCC950, reduced the atherosclerotic plaque burden. Furthermore, IL-1β secretion was completely abrogated in macrophages following treatment with MCC950, suggesting that TET2 deficiency affects NLRP3-mediated IL-1β secretion. Subsequently, these findings have been replicated in other murine models of TET2 deficiency, confirming the association of TET2 deficiency in accelerated atherosclerosis through induction of a proinflammatory state [5]. There has been a suggestion from a small cohort of TET2 deficient atherosclerotic-prone mice (*n*=30) that the response to IL-1β inhibition may be sex-dependent although this needs further exploration [69].

VSMC-derived cells in mouse atherosclerotic plaques are generated by clonal expansion of cells within the vessel wall [70–72]. TET2 is highly expressed in human coronary artery SMCs and, in response to arterial injury, TET2 loss-of-function exacerbates intimal hyperplasia after injury [73]. It has previously been demonstrated that rapamycin induces contractile protein expression in human VSMCs [74]. Rapamycin-induced VSMC differentiation is prevented by TET2 coronary arterial SMC knockdown, whereas TET2 overexpression induces a contractile phenotype suggesting that TET2 acts a regulator of VSMC phenotypic transformation [73].

The endothelium exerts substantial vasoprotective effects. Abnormalities of autophagy homoeostasis, the natural process regulating the removal of unnecessary or damaged cellular components, has been implicated in endothelial cell dysfunction and the development of atherosclerosis, microvascular dysfunction and heart failure. TET2 is an important regulator of autophagy and, following low shear stress, endothelial cell autophagy is reduced via the down-regulation of TET2 [63]. Furthermore, in the ApoE^−/−^ murine model, autophagy is up-regulated by TET2 overexpression and decreased by TET2 silencing [63].

### Jak^V617F^ – preclinical vascular models

The Jak^V617F^ mutation has also been examined in a mouse model of atherosclerosis. Irradiated Ldlr^−/−^ mice were transplanted with bone marrow from either wild type or Jak2^VF617^ mutant mice and subsequently fed a high fat/high cholesterol diet. Despite lower plasma cholesterol levels, the aortic root atherosclerotic lesion size was 1.6-fold higher in Jak2^VF617F^ mice in comparison with WT [75]. Furthermore, Jak2^VF617F^ macrophages had greater expression of pro-inflammatory cytokines and chemokines including, IL-1β, IL-6, IL-18, TNF-α and MCP-1 following challenge with LPS [75]. Even in the absence of LPS stimulation, Jak2^V617F^ mice had higher plasma levels of IL-18 compared with WT controls [75]. However, these Jak2^V617F^ mice developed marked erythrocytosis, thrombocytosis and neutrophilia which is more consistent with a myeloproliferative neoplastic phenotype than CHIP and these confounding effects limit further interpretation. A subsequent experiment examined endothelial function in the common carotid artery of LDLr^−/−^ mice transplanted with Jak2^V617F^ bone marrow cells following constrictive cuff placement across the artery [76]. The carotid arteries of these Jak2^V617F^ mice displayed increased endothelial permeability, reduced endothelial continuity, increased intimal neutrophil extracellular trap accumulation with a subsequent increase in thrombus formation [76]. Treatment with ruxolitinib, a Jak1/2 inhibitor, reduced endothelial cell apoptosis and improved endothelial continuity in Jak2^V617F^ mice [76].

## Heart failure

While heart failure with reduced ejection fraction (HFrEF) is a consequence of impaired left ventricular systolic function (left ventricular ejection fraction [LVEF] <40%), patients with heart failure and preserved ejection fraction (HFpEF; LVEF >50%) reflect a less well understood group in whom ageing and inflammation may play a much larger relative role [77]. Unlike HFrEF, no evidence-based therapies currently exist for the treatment of patients with HFpEF which is more commonly associated with multi-morbidity, myocardial stiffening and macro- and micro-vascular endothelial dysfunction [78–81].

### Human HFrEF and CHIP

The prevalence of CHIP in patients with HF has been assessed in 200 patients with chronic ischaemic HFrEF enrolled in clinical trials of autologous stem cell therapy. In this relatively young cohort (median age 65 years) with a mean LVEF of 31%, CHIP was present in 18.5%. [7]. DNMT3A mutations were observed in 30% of patients and 18% of patients had mutations in TET2. These CHIP mutations were independently associated with heart failure hospitalisation and death (HR 2.1; 95% CI 1.1–4.0) (Table 3) [7]. Notably, the majority of this mortality was attributable to progressive heart failure with only one death occurring as a result of subsequent MI. There was a significant association between clinical outcome and %VAF, further implying a ‘dose effect’ of CHIP [7]. VAF cut-off values of ≥0.73% and ≥1.15% for TET2 and DNMT3A mutations, respectively, were predictive of poorer prognosis [82]. Circulating inflammatory cytokines were not measured in this group but, in a separate very small cohort of six patients with heart failure, the presence of DNMT3A mutation was associated with higher transcription of IL-1β and IL-6 when compared with patients with HF and no DNMT3A mutation [83].

### Human HFpEF and CHIP

HFpEF now accounts for more than 50% of patients with HF, the incidence of which rises substantially with age [81,84]. The pathophysiology of HFpEF remains incompletely understood although structural and functional abnormalities are becoming better defined. Cardiac biopsies obtained from patients with HFpEF reveal structural alterations including cardiomyocyte hypertrophy [85,86] and interstitial fibrosis [85,87–89], while functional changes include impaired myocardial relaxation [90] and increased myocardial stiffness [85,87,88]. Cardiac biopsies also reveal higher levels of myocardial inflammatory cells in patients with HFpEF [91]. Post mortem findings from patients with HFpEF reveal more extensive coronary artery disease, a greater burden of myocardial fibrosis and reduced microvascular density compared with controls without heart failure [92]. Large vessel stiffening is also a feature of vascular ageing and inflammation may, at least in part, contribute to the pathophysiology of HFpEF [93].

Inflammation appears to be more important in the pathophysiology of HFpEF than HFrEF [80]. Circulating concentrations of inflammatory biomarkers including IL-1, CRP and growth differentiation factor 15 are high in HFpEF [94–97] and more so in HFpEF than in HFrEF [98–100]. Network analysis of circulating biomarkers obtained from patients with HFrEF and HFpEF in the BIOlogy Study to TAilored Treatment in Chronic Heart Failure (BIOSTAT-CHF) cohort revealed important differences between the two heart failure phenotypes. In patients with HFrEF, pathways related to cellular growth and metabolism were specifically up-regulated [80] while inflammatory pathways were specifically up-regulated in those with HFpEF [80]. In addition to the inflammatory hypothesis for the aetiology of HFpEF, micro- and macro-vascular disease involving the cardiac, pulmonary and peripheral circulation are highly prevalent in patients with HFpEF [101].

Non-cardiac comorbidities are common in HFpEF, particularly obesity, diabetes, chronic kidney disease and hypertension [79] and the systemic inflammatory state induced by these conditions has recently been shown to be predictive of incident HFpEF but not HFrEF [102]. A novel paradigm to explain the underlying pathogenesis of HFpEF proposes that the systemic inflammatory state induced by these comorbidities induces coronary microvascular endothelial dysfunction. The production of inflammation-induced reactive oxygen species limits the bioavailability of nitric oxide with consequent impairment of cardiomyocyte protein kinase G activity, microvascular ischaemia, fibrosis and left ventricular concentric remodelling [78,98].

Given the associations between HFpEF, vascular dysfunction, inflammation and ageing, we propose that CHIP may be a particularly potent risk factor for the development, progression and potentiation of HFpEF. This hypothesis is yet to be tested directly in humans.

### Preclinical models – heart failure and CHIP

HSC-specific TET2 mutation is associated with the accelerated development of heart failure in murine models of heart failure as a result of left ventricular pressure overload induced by transverse aortic constriction (TAC) and as a consequence of chronic ischaemia induced by ligation of the left anterior descending (LAD) coronary artery [103]. While TAC has been employed as a murine model of HFpEF, after 2–3 weeks TAC results in a reduction in systolic function and progression to HFrEF [104–106]. Following permanent ligation of the LAD, 10% TET2 KO mice had significantly reduced ejection fraction (EF) and this was associated with increased transcription of pro-inflammatory mediators including IL-1β, IL-18, Chemokine (C–X–C motif) ligand 2 (Cxcl2), Chemokine (C–C motif) ligand 2 (Ccl2) and 5 (Ccl5) [103]. Myeloid-specific TET2-deficient mice also had worse cardiac remodelling following LAD ligation with lower LVEF and increased fibrotic area when compared with control mice. Ten percent TET2 KO mice subjected to TAC exhibit marked left ventricular hypertrophy with greater posterior wall thickness and cardiac fibrosis when compared with WT mice. These structural changes were also associated with higher concentrations of circulating IL-1β when compared with control mice [103]. IL-1β cleavage is mediated by the NLRP3 inflammasome, a complex intracellular protein which upon activation, cleaves procaspase-1 protein to functional caspase-1. The primary function of caspase-1 is the conversion of the inactive pro-inflammatory cytokines pro-IL-1β and pro-IL-18 into their active, potently pro-inflammatory states. Over time, TET2 KO mice subjected to TAC also developed systolic impairment. Importantly, administration of MCC950, an NLRP3 inflammasome inhibitor, was associated with significant protection from adverse cardiac remodelling in both models [103].

Bone marrow-specific deletion of TET2 or DNMT3A is associated with cardiac hypertrophy, fibrosis and impaired LV fractional shortening after infusion of angiotensin II in comparison with WT controls [107]. TET2 deletion promoted the expression of IL-1β and IL-6, whereas DNMT3 deletion significantly increased the expression of IL-6 with a trend towards increased IL-1β [107]. Importantly, DNMT3A has been demonstrated to have both direct and indirect roles in maintaining overall cardiomyocyte homeostasis and function [108]. Specifically, DNMT3A^−/−^ engineered human induced pluripotent stem cell-derived cardiomyocytes have up-regulation of pathways involved in cardiac hypertrophy and cardiac proliferation pathways when compared with WT [108]. DNMT3A knock-out also affected contraction kinetics, cell diameter was greater and intracellular lipid accumulation was greater in comparison with the WT [108].

Myeloid-specific Jak2^V617F^ mutation in mice is not associated with abnormalities of peripheral blood count, as would be expected in human CHIP. These animals also do not appear to have abnormalities of cardiac structure or function in the unstressed state [109]. However, following LAD ligation or TAC these mice have greater myocardial macrophage infiltration and concentrations of IL-6 and IL-1β are greater than WT. It has been proposed that Jak2^V617F^ activates the IFN-γ receptor 1 Jak2 signalling transduction pathway (IFNGR1-Jak2-STAT1) resulting in the release of pro-inflammatory cytokines [109]. In the myeloid-specific Jak2^V617F^ model, this mutation was associated with a more substantial deterioration in cardiac function, larger infarct size and increased cardiac fibrosis following TAC/LAD ligation [109]. Furthermore, the adoptive transfer of Jak2^V617F^ bone marrow cells into mice exposed to chronic hypoxia was associated with increased right ventricular systolic pressure and increased muscularisation of pulmonary vessels when compared with control chronically hypoxic mice [110].

While these models have focused upon the investigation of the effects of an exogenous injury or stressor, a recent investigation has attempted to replicate the effects of CHIP in the otherwise ‘unstressed’ state. By transferring TET2-mutant bone marrow cells into mice without prior myeloablative irradiation preconditioning, an attempt was made to replicate the accumulation of somatically abnormal cells over time [111]. In this model, TET2-deficient cardiac macrophages had an overrepresentation of immune response effectors, with specific increases in IL-1β, Ccl17 and IL1-receptor antagonist gene [111]. Concentrations of brain natriuretic peptide (released in response to cardiac pressure overload) were significantly higher in TET2 mutant mice and these animals had greater posterior wall dimension, left ventricular end systolic volume, heart weight and cardiac fibrosis in comparison to control. While LVEF declined slightly, all mice had an LVEF of ≥40% providing evidence that CHIP may be important in the development of HFpEF [111].

## Interplay between CHIP, ageing, inflammation and HFpEF

As outlined, both CHIP and HFpEF are considered to be diseases of the ageing population and both are associated with a systemic pro-inflammatory state (Figure 2). The incidence and prevalence of HFpEF increases sharply with age [112–116], and the mean age of patients with HFpEF in recent cohorts is 72 years [113–115,117–138]. In the context of findings describing the prevalence of CHIP in all-comers, it is reasonable to expect that CHIP is found in at least 10–20% of patients with HFpEF. However, this may be a substantial underestimate. CHIP was found in 27% of patients with chronic ischaemic HFrEF aged between 70 and 79 years [7] and in an elderly population (median age 83 years) with severe aortic stenosis undergoing transcatheter aortic valve implantation (TAVI), the prevalence of CHIP was 33% [8]. In this cohort of patients with severe aortic stenosis, the presence of TET2 or DNMT3A was also associated with an elevated pro-inflammatory subset of circulating leucocytes and conferred a profound increased in mortality even after successful correction of the aortic valve stenosis (HR 3.1 [95% CI: 1.17–8.08]) [8].

In tandem with ageing, the prevalence of comorbidity increases in patients with chronic heart failure [139]. Indeed, nearly half of patients with HFpEF have five or more comorbidities [140]. Many of these comorbidities are associated with a pro-inflammatory state and, furthermore, circulating markers of inflammation are predictive of incident HFpEF [102]. Diabetes occurs in approximately 40% of male patients with HFpEF and 30% of female patients with HFpEF [140]. Diabetes is associated with a two-fold increased risk of developing CHIP and individuals with both diabetes and CHIP have a higher burden of cardiovascular comorbidities than those with diabetes alone [6,141]. It is unclear to what extent these pro-inflammatory comorbidities, considered to be central to the concept of *inflammageing*, are the cause or effect of CHIP, but it is likely that a positive feedback loop is established between them [35].

Younger patients with HFpEF are more likely to be male and have a history of obesity and diabetes [142–144], both of which are strongly associated with chronic low-grade inflammation [142–144]. The presence of CHIP may be of even greater relevance in these younger patients as an indicator of increased epigenetic age. Indeed, the presence of any CHIP mutation confers a 4-year increase in epigenetic age, while CHIP-related TET2 mutation confers a 6-year increase [145]. Deviations from chronological age towards an increased epigenetic age are associated with increased risk of earlier mortality and age related morbidities [146,147].

In 5214 postmenopausal women included in the Womens Health Initiative dataset, the presence of any of the top three CHIP-associated mutations (TET2, DNMT3A and ASXL1) was associated with incident HFpEF but not HFrEF [148]. Women with premature menopause have increased risk of heart failure, stroke, coronary and peripheral arterial disease [149]. Furthermore, systemic markers of inflammation, including CRP, are higher in post-menopausal women than they are in those who are pre-menopausal [150,151]. It is of note that, in women included in the U.K. Biobank and Womens Health Initiative, the prevalence of CHIP was 60% higher in women with premature menopause compared with those without and the presence of CHIP was independently associated with incident coronary artery disease [152]. Whether or not the presence of CHIP and early-onset menopause increases the risk of developing HFpEF is unknown.

## CHIP and personalised cardiovascular management

Historical trials of anti-inflammatory therapy for the treatment of CVD have mainly been disappointing. However, Canakinumab Anti-Inflammatory Thrombosis Outcome Study (CANTOS) has reinvigorated this area and highlights CHIP as a potential biomarker to inform personalised therapy. CANTOS examined the effects of canakinumab, a monoclonal antibody directed against IL-1β, in patients with a history of prior myocardial infarction and elevated CRP. Canakinumab reduced CRP and the incidence of atherosclerotic cardiovascular events was decreased by 15% versus control [153]. Notably, canakinumab also reduced heart failure hospitalisation and heart failure-related mortality by 23% in patients who achieved a CRP level of <2 mg/l [154]. Given the association of CHIP with inflammation and, in particular, the secretion of IL-1β (the immediate upstream precursor to IL-6), CHIP has been proposed as a potential biomarker for personalised therapy with canakinumab and potentially other anti-inflammatory therapies. Indeed, in an exploratory analysis of CANTOS, canakinumab reduced the relative risk of major adverse cardiovascular events by 64% in those with TET2 mutations and by 15% in the treatment overall [155]. Whether or not this impressive effect will also be seen in patients with HF is unknown.

Inzomelid, a novel small-molecule inhibitor of the NLRP3 inflammasome, is currently under clinical investigation for its safety and tolerability in humans (NCT04015076). Whether any potential effect is amplified in patients with CHIP may be a logical future step in its assessment. Recent intriguing data reveal that the sodium-glucose co-transporter 2 (SGLT-2) inhibitor, dapagliflozin, reduces IL-1β via up-regulation of serum β-hydroxybutyrate [156]. Again, the potential benefits of personalisation of SGLT2 inhibitor therapy on the basis of CHIP status is an intriguing but untested hypothesis.

## Conclusion

**Figure 3:**
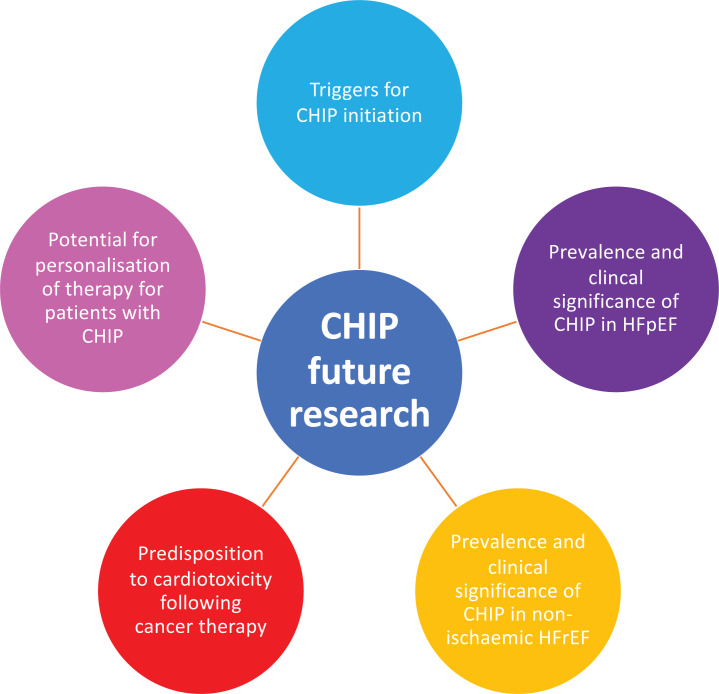
CHIP and future research

While early attention has been paid to the potential role of CHIP in progression to haematological malignancy, it has rapidly become clear that its association with CVD is much stronger. The mechanistic basis to its role in the pathogenesis of atherosclerosis and vascular dysfunction is becoming clearer and further highlights the central role of inflammation in these processes. Preliminary clinical data have highlighted the prevalence of CHIP and its association with poorer outcome in patients with chronic ischaemic HFrEF, while animal models have provided further insight. Given the important intersections among ageing, inflammation and vascular disease in the pathogenesis of HFpEF we believe that CHIP reflects a ripe target for further assessment in this growing group of patients who currently lack evidence-based therapy. Whether CHIP status will allow personalisation of therapy for these patients and others remains an open avenue for future work, with the optimistic aim of harnessing the potential of anti-inflammatory treatments for heart failure (Figure 3).

## Abbreviations

ASXL1: additional sex comb-like 1
CAC: coronary artery calcification
CANTOS: Canakinumab Anti-Inflammatory Thrombosis Outcome Study
CHIP: clonal haematopoiesis of indeterminant potential
CRP: C-reactive protein
CVD: cardiovascular disease
DNMT3: DNA methyltransferase 3
EF: ejection fraction
HFpEF: heart failure with preserved ejection fraction
HFrEF: heart failure with reduced ejection fraction
HSC: haematopoietic stem cell
IFN-γ: interferon-γ
IL: interleukin
KO: knockout
LAD: left anterior descending artery
LDL: low-density lipoprotein
LVEF: left ventricular ejection fraction
MCP-1: monocyte chemoattractant protein 1
NLRP3: nucleotide-binding domain leucine-rich repeat containing receptor 3
TAC: transverse aortic constriction
TET2: ten-eleven-translocation-2
TNF-α: tumour necrosis factor-α
VAF: variant allele frequency
VSMC: vascular smooth muscle cell
5hmC: 5-hydroxymethylcystosine
